# The Biological Roles of *Puccinia striiformis* f. sp. *tritici* Effectors during Infection of Wheat

**DOI:** 10.3390/biom13060889

**Published:** 2023-05-26

**Authors:** Junjuan Wang, Tongtong Chen, Yawen Tang, Sihan Zhang, Mengyao Xu, Meiyan Liu, Jian Zhang, Gary J. Loake, Jihong Jiang

**Affiliations:** 1 School of Life Science, Jiangsu Normal University, Xuzhou 221116, China; 2 School of Biological Sciences, University of Edinburgh, Edinburgh EH9 3JH, UK

**Keywords:** wheat stripe rust (yellow rust) disease, effector, wheat, interaction, plant immunity

## Abstract

*Puccinia striiformis* f. sp. *tritici* (*Pst*) is the causative agent of wheat stripe rust, which can lead to a significant loss in annual wheat yields. Therefore, there is an urgent need for a deeper comprehension of the basic mechanisms underlying *Pst* infection. Effectors are known as the agents that plant pathogens deliver into host tissues to promote infection, typically by interfering with plant physiology and biochemistry. Insights into effector activity can significantly aid the development of future strategies to generate disease-resistant crops. However, the functional analysis of *Pst* effectors is still in its infancy, which hinders our understanding of the molecular mechanisms of the interaction between *Pst* and wheat. In this review, we summarize the potential roles of validated and proposed *Pst* effectors during wheat infection, including proteinaceous effectors, non-coding RNAs (sRNA effectors), and secondary metabolites (SMs effectors). Further, we suggest specific countermeasures against *Pst* pathogenesis and future research directions, which may promote our understanding of *Pst* effector functions during wheat immunity attempts.

## 1. Introduction

*Puccinia striiformis* f. sp. *tritici* (*Pst*) is a member of the large family of rust fungi and caused a most widespread and devastating disease, wheat stripe rust, resulting in significant yield loss on wheat production [1,2], which causes global economic losses of USD 4 billion to USD 5 billion a year [3]. The fungus *Pst* is an obligate biotroph with a complex life cycle. This fungus needs two different hosts to complete its life cycle, and it can produce spermatia, aeciospores, urediniospores, teliospores, and basidiospores [4]. Especially, the urediniospores, commonly used for etiological and evolutionary biology studies, are dikaryotic [4]. *Pst* infects the hosts (cereal crops and grasses) from urediniospore deposition by raindrops or wind onto the leaf surface; then, it infects the host mesophyll cell and forms haustorial mother cells, from which a balloon-shaped feeding structure then forms, known as the haustorium [5]. The rust haustorium is not only the main means for the pathogen to absorb nutrients but also the main field for massive expression and secretion of secretory protein (effector) [6]. However, *Pst* cannot be cultured in vitro, and wheat, as the agronomic host of *Pst*, is not particularly amenable to genetic manipulation due to the relatively difficult genetic transformation and mutant production [7]. Collectively, these factors associated with the pathogen and host hinder research progress from uncovering the detailed biology of *Pst* infection. In recent years, new strains of *Pst* have been continuously emerging, and the development of knowledge about the virulence variation of this fungus could help to obtain new resistant wheat cultivars.

## 2. Plant PTI and ETI

Based on the “zigzag” mode, there are succession steps in the interaction between the plant and pathogen in the plant immune system [8]. Plant transmembrane pattern recognition receptors (PRRs) identify microbial- or pathogen-associated molecular patterns (MAMPs or PAMPs), such as flagellin6, and this recognition results in PAMP-triggered immunity (PTI), which can prevent pathogen colonization. Microbial effectors are thought to have subsequently evolved to suppress this defense system, leading to effector-triggered susceptibility (ETS). In response, plants may have evolved resistance (R) genes, which encode proteins that predominantly recognize the activity of these effector proteins. This can result in effector-triggered immunity (ETI), conferring disease resistance and a hypersensitive cell death response (HR) at the infection site. The effectors that are able to be recognized directly or indirectly by R proteins from the plant are called avirulence (Avr) proteins [9].

### 2.1. Wheat Resistance Genes against Pst

In the gene-for-gene concept [10], a pathogen Avr protein is recognized by a cognate host R protein, leading to the activation of host defense responses [9]. In rust resistance, the yellow rust (*Yr*) genes function as *R* genes compatible with the classical gene-for-gene theory. Among these *Yr* genes, some exhibit resistance that stays effective during its prolonged and widespread use in a favorable environment to the disease, which is considered as durable resistance, and this resistance is non-race-specific [11]. Other *Yr* genes could provide effective resistance against only a few subsets of *Pst* races, and this resistance can be overcome once the new virulent strain emerges, so this kind of resistance is non-durable and race-specific [11]. Currently, although more than 150 temporarily or permanently designated *Yr* genes, together with over 300 quantitative trait loci (QTL), have been identified [12,13,14], only a few *Yr* genes directly linked to resistance have been isolated to date, including *Yr18* [15], *Yr36* [16], *Yr46* [17,18], *Yr5*/*YrSP* [19], *Yr7* [19], *Yr15* [20], *Yr27* [21], *Yr28* [22], and *YrU1* [23] genes. *Yr5*/*YrSP* and *Yr7* genes encode the nucleotide-binding site and leucine-rich repeat (NBS-LRR) proteins with a non-canonical N-terminal zinc-finger BED domain. The *Yr27* gene encodes an intracellular immune receptor, and the *Yr28* gene encodes a typical NBS-LRR protein. At the same time, the *YrU1* gene encodes an NBS-LRR protein that contains an N-terminal ANK and a C-terminal WRKY domain. These genes had been proven race-specific and non-durable. Thus, non-race-specific protection with durable resistance is urgently needed.

*Yr15*, *Yr18*, *Yr36*, and *Yr46* genes, without an NBS-LRR domain, are all members of a non-NBS-LRR class of *R* genes that appear to give long-lasting, non-race-specific resistance to *Pst* [12]. Insights into the molecular mechanisms conveying this resistance have arisen from transcript analysis between the *Yr39* gene (durable resistance) and *Yr5* gene (non-durable resistance), indicating that 14 transcripts that are likely associated with host cell death are expressed and shared by both types of resistance [24]. Meanwhile, some up-regulated genes identified in *Yr39*-mediated but not in the *Yr5*-mediated resistance included *R* genes. Thus, it has been speculated that the *Yr39* gene functions as a master regulator of extra defense-related genes and other R genes, which contributes to its efficiency [24].

At present, the majority of wheat genes (Table 1) that respond to *Pst* infection are resistance-related genes, probably in the downstream of *R* gene-induced defense signaling. Thus, more master *R* genes such as *Yr39* gene need to localize and clone. In recent years, the assembly of the wheat genome has been continuously improved [25], benefiting from development of genomics sequencing, which will accelerate the identification of *Yr* genes.

### 2.2. Pst Avr Gene

Similar to the identification of wheat *R* genes, the identification of *Pst Avr* genes is also far from straightforward. In *Puccinia graminis* f. sp. *tritici* (*Pgt*), the product of the *AvrSr27* gene locus is recognized by the *Sr27* gene in wheat. Moreover, the loss of the *AvrSr27* gene locus can result in *Pgt* strains becoming pathogenic on the wheat cultivars harboring *Sr27* gene resistance; most importantly, the sequences of the *AvrSr27* gene between virulent and avirulent isolate from *Pgt* display divergence [47], and this may be the main determination for the virulence mutation for stem rust fungi. However, rare *Avr* genes have been identified in *Pst*. A total of 127 progeny isolates of *Pst* obtained by selfing a predominant Chinese race, CYR32, on *Berberis aggregate* were obtained and used to testing 25 wheat lines with different *Yr* genes for resistance. Subsequently, the linkage of 10 virulence/avirulence genes was revealed by molecular mapping [48]. A segregating population was obtained via self-fertilizing a *Pst* isolate 12-368 on barberry, and a high-density genetic map consisting of a large number of genome-wide molecular markers was constructed [49]. At the same time, 34 wheat genotypes (*Yr1*, *Yr5*, *Yr6*, *Yr7*, *Yr8*, *Yr9*, *Yr10*, *Yr15*, *Yr17*, *Yr24*, *Yr27*, *Yr32*, *Yr43*, *Yr44*, *YrSP*, *YrTr1*, *YrExp2*, *Yr76*, *Yr2*, *Yr21*, *Yr25*, *Yr26*, *Yr28*, *Yr29*, *Yr31*, *Yr35*, *YrCV*, *YrTr1*, *YrCN19*, *YrA*, *YrAvS*, *Yr45*, *Yr53*, and *Yr64* genes), each harboring a single *Yr* resistance gene, were used to obtain avirulence/virulence phenotypes of *Pst* 12–368 and its progeny isolates [49]. Finally, six *Avr* genes were mapped, including *AvYr8-1*, *AvYr27*, *AvYr44*, *AvYr7*, *AvYr43*, and *AvYrExp2* genes [49]. Most importantly, this research demonstrated that the inheritance of avirulence/virulence in *Pst* is isolate-dependent, indicating the complex interaction between *Pst* and wheat. The development of molecular markers for *Avr* genes is beneficial for identifying the location of *Avr* genes. A total of 157 *Pst* isolates representing 126 races with diverse virulence spectra were selected and genotyped using 209 highly expressed secreted protein gene (SP)-based single-nucleotide polymorphism (SP-SNP) markers via association analysis [50]. It was found that 19 SP-SNP markers had significant associations with 12 *Avr* genes: *AvYrSP*, *AvYr1*, *AvYr6*, *AvYr7*, *AvYr9*, *AvYr10*, *AvYr24*, *AvYr27*, *AvYr76*, *AvYr32*, *AvYr43*, and *AvYr44* [50]. Advances in whole-genome sequencing of *Pst* will lead to the development of highly diagnostic SP-SNP markers, which could be used to detect more *Avr* genes and clone them, uncovering the virulence variation. Although the *Avr* genes were mapped, the progress of cloning these genes is slow. Therefore, construction of the *Pst* population with near isogenic lines or monogenic lines is crucial for cloning of the *Avr* effector.

### 2.3. Pst Effectors

Currently, many effectors have been identified during numerous plant–pathogen interactions [47,51]. To date, the majority of the effector research has focused almost exclusively on secreted proteins [52,53]. The small non-coding RNAs (sRNA effectors) and fungal secondary metabolites (SMs effectors), which were collectively defined as non-proteinaceous effectors (NPEs) [54], have been even less well characterized in *Pst*. An ever-increasing effector repertoire is being uncovered for *Pst* due to a series of genome sequence datasets [26,55,56,57]. Thus, in this review, we detail *Pst* proteinaceous, sRNA, and SMs effectors that are confirmed or proposed to interfere with wheat immunity, which would help us better understand the biological roles of *Pst* and bring enlightenment to the prevention and control strategy of wheat stripe rust.

#### 2.3.1. Proteinaceous Effectors

##### Proteinaceous Effectors with Unknown Sequence May Exhibit Structure Specificities

To date, more than two thousand proteinaceous effectors have been predicted for *Pst*. Some proteinaceous effectors of *Pst* without a known sequence domain may also perturb wheat immunity. For instance, PEC6, with a non-typical domain localized within the nucleus and cytosol, can suppress PTI and interact with a wheat adenosine kinase (ADK), further controlling wheat leaf growth [27]. In addition, overexpression of PSTha5a23, which lacks any known sequence motifs and is localized to the cytosol, can suppress PTI-associated callose deposition and significantly enhance *Pst* virulence in wheat [29]. PstCEP1 contains only four cysteine (Cys) residues and suppresses PTI and ETI [30]. Furthermore, two *Pst* effectors, namely Pst_4 and Pst_5, lacking chloroplast transit peptides, have low sequence similarities and can interact with TaISP (an iron-sulfur subunit of cytochrome b6-f complex in wheat), which is a chloroplast protein encoded by a nuclear gene, further leading to disrupting chloroplast protein sorting, reducing host accumulation of reactive oxygen species (ROS), and promoting fungal pathogenicity [31]. Recently, PstSIE1, an effector lacking any known sequence motifs, was discovered to compete for TaSGT1 binding. Consequently, this disrupted the formation of the TaRAR1–TaSGT1 subcomplex, a chaperone complex that acts as a core modulator in plant immunity, promoting pathogenesis [32].

We posit that a structural biology approach, i.e., computational structural genomics based on template-free modeling by TrRosetta, might identify additional *Pst* effectors. This approach has been employed to predict the secretome of the destructive fungal pathogen *Magnaport oryzae*. From 1854 secreted proteins, the folds of 1295 proteins (70%) were predicted. Informatively, 514 folds were missed by homology modeling [58]. Therefore, computational structural genomics could be employed to interrogate the *Pst* secretome, potentially promoting structural prediction and identification.

##### Proteinaceous Effectors with Structural Features May Contribute to Diverse Functions

Most fungal effectors do not contain similar sequences or motifs to other proteins. In *Pst*, a wheat NPR1 interactor (PNPi) contains the sequence RSLL-DEEP, which is similar to the RxLR-dEER motif commonly observed in oomycetes [59]. Moreover, this protein interacts with the NPR1/NIM1-like domain of NPR1, suppressing the wheat’s systemic acquired resistance response, by sharing the conserved DPBB_1 (Rare lipoprotein A (RlpA)-like double-psi beta-barrel) domain at the C-terminal region with PNPi homologs from *Pgt*, *Puccinia striiformis* f. sp. *hordei* (*Psh*), and *P*. *triticina* (*Pt*). Recently, it was found that PNPi could also target the CAPE1 region of TaPR1 either in the apoplastic space or the extra-haustorial matrix, which may contribute to suppressing the wheat serine/threonine-protein kinase D6PKL1(TaAdi3) and TaPR7 [33]. Thus, we speculate that the RSLL-DEEP and DPBB_1 domains are probably the key motifs interacting with target genes products in wheat, and these domains may act as a host cell translocation motif in *Pst*. Sequence analysis showed that the *Pst* effector Pst18363, an orthologue of *Uromyces fabae* effector Uf-RTP1, shares two aggregation domains, seven highly conserved β strands, and four conserved cysteines in the C-terminus with other RTP1p homologs [34,60]. The β-aggregation domains and four conserved cysteines of Uf-RTP1 are associated with the formation of filamentous structures and inhibitory activity of cysteine protease, respectively [61,62]. Therefore, we predict that the Pst18363 is involved in the ability to form hyphae and inhibit cysteine protease. Furthermore, Pst18363 was found to interact with wheat Nudix hydrolase 23 (TaNUDX23) and enhance its hydrolase stability, further suppressing reactive oxygen species (ROS) accumulation and leading to the facilitation of *Pst* infection [34]. These findings indicate that some *Pst* effectors may manipulate host immunity by different signal mechanisms by sharing similar known sequences/domains with those from other fungi.

##### Proteinaceous Effectors Are Rich in Specific Amino Acids Representing Specific Functions

Notably, in some cases, some proteinaceous effectors are rich in a certain amino acid (aa). An additional series of fungal effectors is cysteine-rich. In this case, PSTG_14695 [35] and PstSCR1 [36], two cysteine-rich proteins from *Pst*, can suppress and induce plant defense response, respectively. Multiple cysteine residues in fungal effectors allow the formation of the intramolecular disulfide bond, maintaining protein stability [63]. Thus, we speculate that abundant cysteine residues within *Pst* proteinaceous effectors are associated with their cognate stability.

PstGSRE1 [37] and PstGSRE4 [38] are good examples in the context of glycine-serine-rich effectors. The former contains a glycine-serine-rich motif (m9) and interacts with a wheat transcription factor, TaLOL2 [37], while the latter lacks the m9 motif and interacts with a wheat copper-zinc superoxide dismutase (SOD), i.e., TaCZSOD2, but not TaLOL2 [38]. Further, both proteins can suppress ROS-mediated cell death, resulting in the suppression of host immunity. Further, a serine-rich effector, Pst27791, can suppress ROS accumulation, and the virulence of this effector was mediated by interaction with wheat’s rapidly accelerated fibrosarcoma (Raf)-like kinase TaRaf46 [39]. It was discovered that some *M. oryzae* effectors rich in glycine/serine participate in regulating the activity of a variety of antioxidant enzymes, which lowers the level of ROS in the host, leading to a decrease in the host immune response [64,65]. Thus, we speculate that the glycine/serine-rich effectors of *Pst* can specifically regulate ROS accumulation and associated signal transduction in wheat.

Recently, it was discovered that Pst_A23, an arginine-rich *Pst* effector protein that localizes to host nuclear speckles (nuclear regions enriched in splicing factors), functions as a “splicing” effector by directly binding to a target *cis*-element within both *TaXa21-H* and *TaWRKY53* and regulating the pre-mRNA splicing of these two wheat genes, thereby impairing wheat resistance to *Pst* [40]. Some cell-penetrating peptides (CPPs) with multiple arginine residues could facilitate protein transport across membranes [66], indicating that arginine residues may promote effector transport from the apoplast to the cytoplasm in the target plant cell. However, in-depth sequence analysis and cognate biological interrogation will still be needed in the future to verify the function of the enriched specific amino acid residues within effectors, and the resulting information may contribute to an increased understanding of *Pst* effector biology.

##### Proteinaceous Effectors Exhibiting Diversity in Primary AASequences but Conserved Tertiary Structural Motifs

An additional class of fungal proteinaceous effectors exhibits a generally high diversity amongst primary aa sequences while exhibiting conserved tertiary structural motifs. For example, ToxB, an effector from the wheat tan spot pathogen *Pyrenophora tritici-repentis*, shares six β-sandwich structures with AvrPiz-t, another effector from *M. oryzae*, although their primary aa sequences are unrelated [67]. These proteinaceous effectors have been termed MAX-effectors (Magnaporthe Avrs and ToxB-like), and they are expressed specifically at an early infection stage, indicating their important functions during biotrophic host colonization. Although numerous effectors from *Pst* have been identified (Table 1), their three-dimensional structures have not been routinely analyzed. However, Pst-1374 was reported to exhibit that majority of the structures crimp, and the trifluoroethanol can stabilize its structure, increasing the ratio of α-helix. Additionally, Pst-1374 is able to form polymers on its own and depolymerize when interacting with the wheat thiogen-reducing protein (TaTrxm), which localizes to the chloroplast and controls numerous Calvin cycle enzymes [41]. These properties may be crucial functional adaptations of this effector. Although many sequencing studies have obtained the primary sequences of *Pst* proteins [68], the main tertiary structural motifs such as 6 β-sandwich structures (contributing to biotrophic colonization) are needed to elucidate the secreted proteins in *Pst*. Computational structural genomics based on template-free modeling would provide a novel approach to tackle this problem [58], which may show structural conservation among *Pst* effectors.

Interestingly, most of the identified wheat targets of *Pst* effectors are defense-related proteins that are key components of the host immune response. However, it has been reported that a susceptibility gene, wheat receptor-like cytoplasmic kinase TaPsIPK1, is bound by *Pst* effector PsSpg1. This interaction can enhance the kinase activity and nuclear entry of TaPsIPK1, promoting *Pst* pathogenesis [42].

#### 2.3.2. Small RNA Effectors

Small RNAs (sRNAs), which are short non-coding RNA molecules and include small interfering RNA (siRNA) and microRNA (miRNA), can trigger the silencing of target gene expression at the transcriptional and posttranscriptional levels [69]. siRNA originates from complementary long double-stranded RNAs (dsRNAs) [70], and miRNA is generated from a single-stranded precursor with self-complementarity [70]. The first documented role for RNA interference (RNAi) was discovered in *Neurospora crassa* [71]. Subsequently, sRNA from other fungal species were found to regulate gene function [72,73]. Pathogen-derived sRNAs and the associated RNAi machinery could contribute to pathogen virulence [70,74]. In this context, *Botrytis cinerea* small RNAs (Bc-siRNAs) are virulence effectors interfering with plant immunity. Bc-siRNA target genes encode a cell wall-associated kinase (WAK), *Arabidopsis* mitogen-activated protein kinase MPK1 and 2, a *Peroxiredoxin* (*PRXIIF*), and the tomato MPK-kinase kinase 4 (MAPKKK4) that positively regulates plant immunity. All these targets are suppressed during attempted *Botrytis cinerea* infection [75]. Further, this suppression was also found in transgenic plants overexpressing Bc-sRNAs. Moreover, Bc-sRNAs can target the *Arabidopsis* Argonaute (AGO) protein, the main executer of sRNA-mediated post-translational gene silencing (PTGS), by silencing host genes employing host gene-silencing machinery. The emerging evidence suggests that Bc-siRNAs suppress host plant immune responses, enhancing pathogen infection.

The sRNAs of *M. oryzae* regulate subsets of mRNAs post-transcriptionally, including *ACE1*, which encodes a proteinaceous effector. The expression of ACE1 can be activated by appressorium-mediated penetration [74].

Identification of the roles of sRNAs within biotrophic plant fungal pathogens such as *Pst* is only recently emerging. sRNA from *Pst* (PST-100)-infected wheat flag leaves were identified and interrogated by high-throughput sequencing, which revealed an abundance of 20–22 nt sequences, with a preference for uracil at the 5′ position. sRNA-target gene prediction was divided into several classes: fungal target genes were rich in kinases and small secreted proteins, while wheat gene targets included known plant resistance gene homologs [72]. Further, the *Pst* sRNAs were hypothesized to be processed in a Dicer-dependent manner. A novel microRNA-like RNA from *Pst* (PstmilR1) was also found to target the wheat *Pathogenesis-related 2* (*PR2*) gene, triggering gene silencing by cross-kingdom RNAi and thus suppressing wheat defenses against *Pst* infection [46].

Moreover, the biology of sRNA in *P. triticina* was investigated following *P. triticina* pathotype 77–5 infection of wheat leaves. This analysis isolated about 1–1.28 million potential sRNAs, including two microRNA-like small RNA (mil-RNAs) candidates [76]. It was predicted that numerous targets of sRNAs in *P. triticina* were repetitive elements, while in wheat, sRNA targets included genes related to disease resistance, ROS pathways, metabolic processes, PCD regulation, and transcription factor function [76]. Three microRNA-like RNA molecules (mil-RNAs) were characterized in *P. triticina*, of which PTmilR2* was a MAP kinase that was demonstrated by degradome mapping and qRT-PCR [76]. Compared with other fungi, the regulatory mechanism of sRNA in *Puccinia* species has not been explored in depth. How sRNA function directly or indirectly regulates pathogenicity and plant immunity by the cross-kingdom RNAi machinery still needs to be investigated carefully.

#### 2.3.3. SMs Effectors

Fungal secondary metabolites (SMs) are commonly divided into four main chemical classes: terpenoids, polyketides, shikimic-acid-derived compounds, and non-ribosomal peptides [77]. 1,16-hexadecanediol and 1,16-hexadecanedial from *M. grisea* were found to be inducers for fungal germination and appressorium formation [78], further supporting the infection process. The necrotrophic phytopathogenic fungus *B. cinerea* has a wide host range [79]. Some phytotoxic metabolites have been characterized in this fungus. Of these, the sesquiterpene-derived phytotoxin botrydial has been implicated in virulence, as this molecule can promote fungal penetration and colonization in plants [80]. In contrast, biotrophic fungi are not known to produce a significant repertoire of SMs [77]. The plant biotrophic pathogen *Cladosporium fulvum* (*Passalora fulva*), which can infect tomatoes and grows extracellularly adjacent to host mesophyll cells, synthesizes cladofulvin, which is currently the only known SM produced by this fungal pathogen [81,82]. However, to date, no correlation has been established between this SM and the pathogenesis process of Solanaceae species by *C. fulvum* [81]. Therefore, based on the available data to date, the relative absence of SM biosynthesis is related to biotrophy [83]. Counterintuitively, the biotrophic *C. fulvum* has twice the number of key SM genes compared to the closely related hemibiotrophic fungus *Dothistroma septosporum* (teleomorph *Mycosphaerellapini*) [84]. Therefore, it is probable that the biotrophic lifestyle has a special mechanism that involves downregulating the expression of a significant number of genes linked to SM biosynthetic pathways rather than reducing the capacity to produce SM [84].

For *Pst*, there is no report about any SMs produced by this biotrophic pathogen, which may be correlated with the lifestyle of this fungus. Omics technologies bring hope to the exploration of SMs from *Pst* during infection. Overall, 33 and 29 SMs have been annotated from *Psh* (93TX-2) and *Pst* (93–210), respectively, by genomic technologies [56]. By transcriptomic analysis, six genes encoding terpenoids and polyketides in differentially expressed genes (DEGs) were predicated, which were involved in wheat durable high-temperature seedling-plant (HTSP) resistance in cultivar Xiaoyan6 [84]. This indicates that terpenoids and polyketides may be linked to the *Pst* interaction with wheat. However, compared with proteinaceous effectors, the number of SM effectors in *Pst* is limited. Thus, in order to assist in increasing the number of SMs found in *Pst* pathogenesis in particular and other biotrophic diseases in general, increased application of omics methods is necessary.

**Figure 1:**
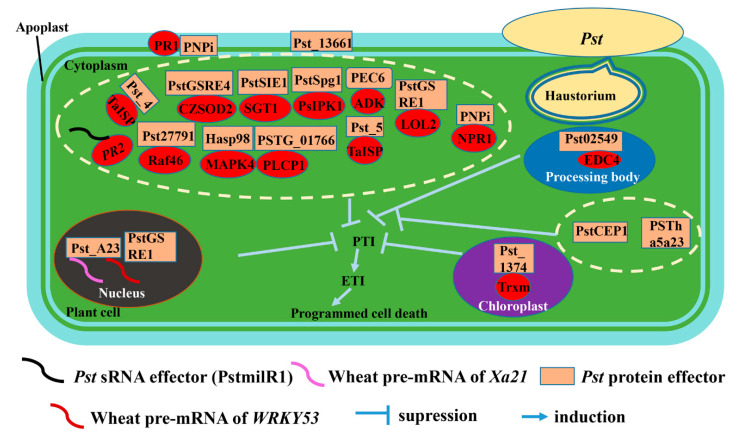
Model of different types of *Pst* effector interacting with wheat target proteins. *Pst*, *Puccinia striiformis* f. sp. *tritici*; PTI, pathogen-associated molecular-patterns-triggered immunity; ETI, effector-triggered immunity; PR1, pathogenesis-related protein 1; *PR2*, the gene of pathogenesis-related protein 2; TaISP, wheat cytochrome b6-f complex iron-sulfur subunit; CZSOD2, wheat copper-zinc superoxide dismutase; Raf46, wheat rapidly accelerated fibrosarcoma (Raf)-like kinase; SGT1, suppressor of the G2 allele of S-phase kinase-associated protein 1 (skp1); MAPK4, wheat mitogen-activated protein kinase 4; PsIPK1, wheat receptor-like cytoplasmic kinase gene; PLCP1, phosphorylates papain-like cysteine protease 1; ADK, wheat adenosine kinase; NPR1, wheat non-expresser of pathogenesis-related genes 1; LOL2, the reactive oxygen species (ROS)-associated transcription factor; EDC4, enhancer of mRNA decapping protein 4; Trxm, thiogen-reducing protein.

In Figure 1, an overview of different effectors from *Pst* is depicted. Significant in-depth research is still required to confirm or reject the proposed models. Effectors play an important role in the interaction system between pathogen and plant. Substantial evidence shows that effectors can interfere with plants from multiple pathways and promote the infection of the pathogen, while plants can recognize them and trigger plant immunity [85]. Currently, the majority of research is focused on the regulation of host immune mechanisms, while the transportation mechanism of effectors has been rarely explored in rust fungi. An effector, namely Uf-RTP1 from *Uromyces fabae*, was shown to transport into plant cells by immunolabeling test [60]. Fortunately, the transportation study of effectors in *M. grisea* could give us enlightenment. In a newly entered rice cell, the *M. grisea* hypha produced by appressorium differentiates from filamentous to bulbous invasive hypha (IH), which is along with the formation of the biotrophic interfacial complex (BIC) [86], which is at the tip of the initially filamentous hyphae and then is left behind the bulbous IH. Additionally, the effectors are firstly secreted into BICs when the pathogen enters a new rice cell. In *M. grisea*, numbers of effectors were observed to have preferentially secreted into BIC and then entered into rice cytoplasm [87,88], which was considered a success for the effectors transporting from blast fungus to rice cytoplasm. For example, the effector proteins of blast fungus, i.e., PWL2 and BAS1, were localized into BIC and then translocated into the rice cytoplasm by fluorescence observation [86]. More interestingly, PWL2 and BAS1 proteins reached the rice cytoplasm and then moved into uninvaded neighbors, realizing cell-to-cell movement [86]. These effectors that are secreted through the BIC and translocated into host cells belong to cytoplasmic effectors. Another kind of *M. grisea* effector that is commonly secreted by the conventional endoplasmic reticulum to Golgi routing is called apoplastic effectors [89]. An effector protein named Osp24, which is from the wheat pathogen *Fusarium graminearum* (causes wheat Fusarium head blight), was observed from the nucleus of wheat coleoptile cells at the inoculation site, and it was speculated that the translocation of Osp24 into wheat cells is through BIC-like structures formed by *F. graminearum* [90]. Therefore, it is worth noting that the interacting system between blast fungus and rice is a good model to research the secretion, translocation, and cell-to-cell movement for *Pst* effectors. Furthermore, whether the effectors of *Pst* have two similar paths for secretion, similar to blast fungi, or some other unknown path to secrete needs further research.

Due to the especially obligate biotrophic parasite, the genetic transformation of *Pst* is difficult. Most importantly, it is possible that genome rearrangement between the parental and reference isolates occurred because the genetic map of *Pst* was created by heterozygous dikaryotic (having two unfused nuclei in a cell) urediniospores. If markers from the *Avr* locus were located close to one another in the genetic map, but their locations were not linearly correlated, this would lead to inconsistencies. Thus, a deep investigation of sexual recombination events related to the *Pst* genome is needed. Moreover, the *Pst* population with similar genetic background and diverse virulence needs to be constructed, and some mutation methods such as ultraviolet ray (UV), EMS, temperature, UV + EMS, and UV + temperature could be used to produce the mutated isolates of *Pst*. In addition, an improved and haplotype-solved reference genome is required, which could be generated by long-read sequencing technologies such as bacterial artificial chromosome sequencing and high-fidelity (HiFi) reads. Recently, a new *Pst* assembly 134 E16 A+ 17+ 33+ was released using nanopore sequencing [68]. In addition, the more race genome architectures of *Pst* that are constructed, the more *Avr* genes will be isolated. Consequently, understanding the rapid evolution of *Pst* virulence and identifying additional *Pst* effectors will be significantly accelerated.

## Figures and Tables

**Table 1:** The features of determined effectors from *Pst*.

Candidate Effectors	Subcellular Localizations	Known Domain/Amino Acid Enrichment	Host Targets	References
Protein				
PST02549	Processing bodies	Nd	Enhancer of mRNA decapping protein 4 (edc4)	[26]
PEC6 Pst_12806	Nucleus Chloroplast	Nd Chloroplast-targeting sequence	Adenosine kinases Taisp	[27,28]
PSTha5a23	Cytosol	Nd	Nd	[29]
PstCEP1	Cytoplasm	Nd	Nd	[30]
PSTG_01766	Cytoplasm	Nd	Taplcp1	[30]
Pst_4	Cytoplasm	Nd	Cytochrome b6–f complex iron–sulfur subunit	[31]
Pst_5	Cytoplasm	Nd	Cytochrome b6–f complex iron–sulfur subunit	[31]
PstSIE1	Cytoplasm	Nd	Tasgt1	[32]
PNPi	Apoplast	Rxlr-deer, dpbb_1	Npr1, pr1	[33]
Pst18363	Nd	Two aggregation domains, seven highly conserved β strands	Nudix hydrolase 23	[34]
PSTG_14695	Nd	Cysteine-rich	Nd	[35]
PstSCR1	Nd	Cysteine-rich	Nd	[36]
PstGSRE1	Nucleus	Glycine- and serine-rich	Transcription factor (lol2)	[37]
PstGSRE4	Cytoplasm	Glycine- and serine-rich	Taczsod2	[38]
Pst27791	Cytoplasm and nucleus	Serine-rich	Taraf46	[39]
Pst_A23	Nuclear speckles	Arginine-rich	Cis-element of xa21-h and wrky53	[40]
Pst-1374	Chloroplast	Nd	Thiogen-reducing protein (trxm)	[41]
PsSpg1	Cytoplasm	Nd	Tapsipk1	[42]
Hasp98	Cytoplasm	Nd	Tamapk4	[43]
Pst_13661	Apoplast	Polysaccharide deacetylase	Nd	[44]
PstCFEM1	Nd	Common in fungal extracellular membrane domain	Nd	[45]
Small RNA				
PstmilR1	Cytoplasm	Nd	Pr2	[46]

Nd: not determined.

## Data Availability

Not applicable.
